# Organotropic metastasis in colorectal cancer: integrating molecular pathways with therapeutic opportunities

**DOI:** 10.3389/fimmu.2025.1686071

**Published:** 2025-12-02

**Authors:** Hanhui Jing, Yan Gao, Zhongsheng Sun, Ying Li, Jin Wang, Liangliang Zhang, Shanglong Liu

**Affiliations:** 1 Department of Gastrointestinal Surgery, The Affiliated Hospital of Qingdao University, Qingdao, Shandong, China; 2 Department of Blood Transfusion, The Affiliated Hospital of Qingdao University, Qingdao, Shandong, China; 3 Department of Spine Surgery, The Affiliated Hospital of Qingdao University, Qingdao, Shandong, China; 4 Department of Comprehensive Surgery, Kashi Prefecture Hospital of Traditional Chinese Medicine, Kashi, Xinjiang, China

**Keywords:** colorectal cancer, metastasis, influencing factors, artificial intelligence, therapeutic opportunities

## Abstract

Colorectal cancer (CRC), characterized by high incidence and mortality rates, is an aggressive malignancy that significantly burdens public health. Metastasis represents the principal factor contributing to treatment failure in CRC patients, largely due to limited comprehension of the underlying mechanisms governing this phenomenon. CRC metastasis involves multiple factors, including dynamics within the tumor microenvironment (TME), epithelial–mesenchymal transition (EMT), and the dissemination of cancer cells through the circulatory and lymphatic systems. These mechanisms are regulated by complex molecular interactions. A deeper understanding of the metastatic processes and the identification of viable therapeutic targets could substantially advance innovative clinical interventions. This review highlights key contributors to CRC metastasis, integrates relevant molecular mechanisms with distinct patterns of organ-specific spread, and emphasizes the latest advancements in this field. Additionally, it explores experimental models of CRC and metastasis, provides mechanistic insights, and addresses challenges in the clinical management of metastatic CRC. This article aims to facilitate future research and highlight promising therapeutic opportunities for clinical translation.

## 1 Background

Metastasis, characterized by the dissemination of cancer cells to distant organs from their original site, signifies the ultimate and most lethal stage of cancer advancement (1). Most cancer-related deaths are linked to metastatic disease, rather than the original tumors (2). Localized interventions like surgical procedures and radiation therapy can yield positive outcomes for primary tumors; however, metastatic cancer represents a systemic disease that impacts various organs throughout the body (3). This may occur through direct colonization, which disrupts organ function, or by altering organ metabolism through modified secretomes, ultimately leading to death (4). Consequently, systemic therapies, including screening, chemotherapy, targeted therapies, and immunotherapy, are essential for preventing and managing tumor metastasis (5, 6). Recent collaborative research initiatives have achieved notable advances in enhancing cancer treatment outcomes.

Advances in basic research and clinical oncology are essential for further improving the detection and management of metastatic cancers (7, 8). Historically, there has been an unprecedented collaboration between cancer biologists and clinical researchers. Recently, the expanded use of artificial intelligence in the medical field, along with technological advancements, has facilitated the rapid accumulation of metastatic indicator data, annotated with information on disease progression and drug responses (9). Clinical trials are increasingly incorporating predictive models for pre-treatment assessment and post-operative metastasis management.

These strategies enable researchers to efficiently identify biomarkers of therapeutic response. The comprehensive datasets generated through these approaches offer valuable insights into metastatic mechanisms, which are subsequently validated in animal models (10, 11). Consequently, the dynamic interplay between preclinical and clinical research is deepening our understanding of metastatic biology and driving the development of innovative therapies (12).

In this review article, we focus on organ-specific targeting processes in cancer, predictive models, and therapeutic strategies.

## 2 Factors influencing CRC metastasis

### 2.1 CRC microenvironment

**Figure 1:**
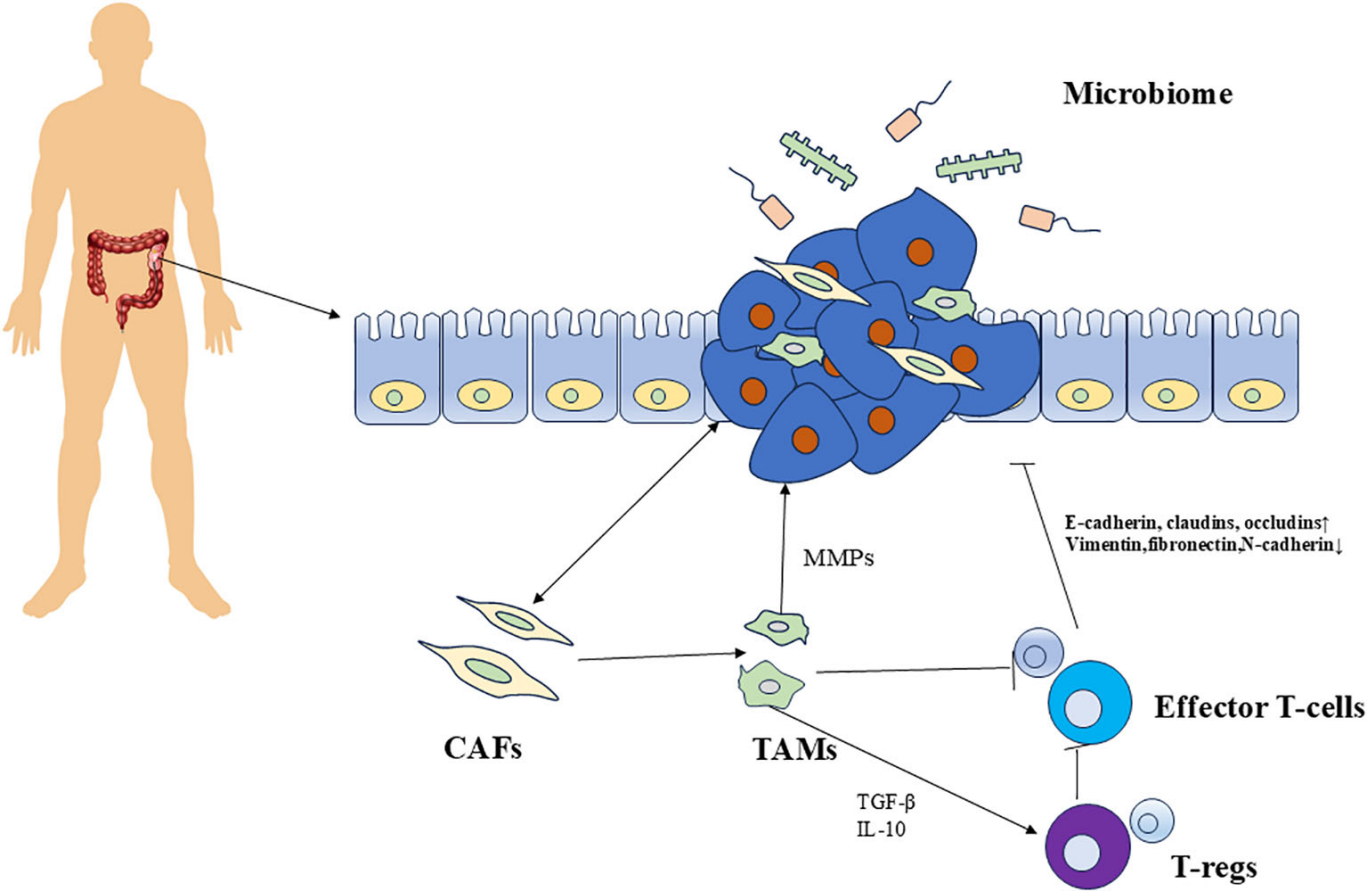
Factors influencing CRC metastasis. The TME is composed of tumor cells, resident host cells (colonic epithelium), immune cells, endothelial cells, neurons, adipocytes, secretory factors, and ECM. Interactions between TME components and tumor cells, as well as between the gut microbiome and tumor cells, regulate tumor invasion and metastasis. MMP, Matrix Metalloproteinases; CAF, Cancer-Associated Fibroblasts; TAM, Tumor-Associated Macrophages; T-reg, Regulatory T Cells; TGF-β, Transforming Growth Factor Beta. Arrows pointing to the tumor indicate promoting effects.

The CRC microenvironment consists of CRC cells, stromal cells (SCs), and the extracellular matrix (ECM), which together facilitate the growth and proliferation of tumor cells (13, 14). SCs comprise fibroblasts, immune cells, and specialized vascular endothelial cells (VECs). In 1889, Stephen Paget introduced the “seed and soil” theory, emphasizing the interaction between tumor cells and the TME and forming the basis for modern research in this field (15, 16). The interplay between tumor cells and the TME is crucial for organ-specific metastasis and the establishment of metastatic niches. Furthermore, research indicates a positive correlation between tumor aggressiveness and the density of SCs present in the microenvironment. A comprehensive exploration of the CRC microenvironment will certainly facilitate the advancement of targeted therapies focused on the TME, promoting the innovation of new anticancer agents and treatment approaches (**Figure 1**).

#### 2.1.1 Tumor cell metabolism in the CRC microenvironment

The TME is crucial in facilitating tumor invasion and metastasis via multiple mechanisms. Tumor cells frequently engage in aerobic glycolysis, leading to significant lactate production that contributes to the acidification of the TME (17, 18). In numerous tumors, a metabolic alteration takes place that favors glycolysis instead of oxidative phosphorylation (OXPHOS), resulting in heightened glucose uptake and the upregulation of glucose transporters, a phenomenon referred to as the Warburg effect (19, 20). Glucose functions as the main energy source for tumor cells; however, the capacity to exploit alternative energy substrates is associated with more aggressive and migratory cancer characteristics (21). Recent findings indicate that metastatic cells exhibit dynamic metabolic changes, allowing them to adapt to the shifting microenvironment throughout the metastatic journey (22, 23).

Metabolites like pyruvate and lactate play a crucial role in augmenting the invasive and migratory abilities of cancer cells (24). Pyruvate is introduced into the tricarboxylic acid (TCA) cycle through the action of pyruvate carboxylase (PC), which facilitates an invasive phenotype by enhancing cellular motility. Chuang et al. demonstrated in a mouse model that metastatic cell lines exhibit reduced mitochondrial membrane potential and impaired mitochondrial function compared with non-metastatic primary tumors (25). Although reduced mitochondrial function can have diverse consequences, evidence indicates that metastatic cancer cells markedly increase both lactate production and its utilization. Wei et al. observed elevated concentrations of lactate dehydrogenase (LDH) and serine lactate aminotransferase (SLA) in patients with metastatic CRC when compared to individuals with non-metastatic disease (26). During colonization of target organs, metastatic cells exploit extracellular pyruvate to remodel the ECM, thereby creating a microenvironment conducive to their proliferation. Increased concentrations of pyruvate facilitate the transamination processes involving glutamate and pyruvate, while also boosting the activity of collagen prolyl-4-hydroxylase (P4HA) (27). This enzyme plays a vital role in the remodeling of the ECM within the pre-metastatic niche.

Studies have shown that cancer cells exposed to hypoxia *in vivo* exhibit increased invasiveness, while the acidic microenvironment of solid tumors—driven by elevated glycolytic activity—also promotes invasion and metastasis. Estrella et al. reported that tumor invasion most frequently occurs in regions with the lowest pH, with no invasion observed in adjacent areas maintaining near-physiological pH levels (28, 29). Furthermore, intraperitoneal lactate administration in mice has been shown to induce the formation of lung metastases (30).

Hypoxia activates glycolytic pathways, resulting in lactate accumulation within the TME and subsequent pH reduction, which further alters cancer cell behavior (31). As a result, cancer cells adapt their metabolic programs to these environmental pressures, thereby promoting invasion and metastasis.

#### 2.1.2 Role of SCs in the CRC microenvironment

Numerous studies have demonstrated that SCs within the TME play essential roles in the initiation, progression, invasion, and metastasis of CRC. The relationship between SCs and tumor cells is complex, with bidirectional interactions that shape a microenvironment favorable to metastatic dissemination.

Cancer-associated fibroblasts (CAFs) have emerged as critical contributors to CRC progression through a variety of mechanisms. Sugai et al. demonstrated that fibroblast activation protein-1 (FAP-1), expressed by CAFs, promotes lymph node metastasis (LNM) and tumor growth (32). Another study demonstrated that CAFs drive CRC progression via the Wnt signaling pathway, particularly Wnt2, which is secreted in a paracrine manner and facilitates CRC-CAF interactions (33, 34). CAF-derived Wnt2 promotes the metastasis and invasion of CRC by stimulating the suppressor of cytokine signaling-3 (SOCS3) in dendritic cell precursors. This activation leads to the inhibition of the p-Janus kinase 2 (JAK2)/p-STAT3 (Tyr705) pathway, ultimately suppressing dendritic cell differentiation (35).

Macrophages that infiltrate tumor tissues or populate the solid TME are known as tumor-associated macrophages (TAMs) (36, 37). Immune cells play a pivotal role in regulating immune responses during tumor growth, angiogenesis, invasion, and migration, thereby facilitating the immune evasion strategies employed by cancer cells (38, 39). Tumor associated macrophages (TAMs) are broadly classified into two subtypes: anti-tumor M1 and pro-tumor M2 macrophages. The transition between these phenotypes, known as macrophage polarization, is driven by diverse microenvironmental signals (36, 40).

The polarization of TAM is modulated by a range of cytokines, chemokines, growth factors, and additional signals generated by both tumor and SCs (41, 42). Among these, colony-stimulating factor-1 (CSF-1) and C-C motif ligand 2 (CCL2) stand out as particularly significant (43, 44). In tumor implantation models, CSF-1 reduction decreases macrophage density and significantly suppresses metastasis (45–48). Moreover, high CSF-1 expression at the tumor periphery has been associated with an increased risk of metastatic spread (49). Additional factors, including IL-4, IL-6, IL-13, CCL7, and CCL8, are upregulated in tumors and contribute to the recruitment and polarization of TAMs (50–52). Within the TME, hypoxia and acidic conditions further promote TAM migration, supporting tumor cell adaptation to nutrient deprivation and shaping a microenvironment favorable to tumor progression (53, 54). Recent studies highlight the role of TAM in the degradation of the ECM (55). TAMs secrete hydrolytic enzymes, including cathepsins and serine proteases, which facilitate ECM remodeling (56, 57). Evidence indicates that M2 macrophages enhance tumor aggressiveness through the secretion of chitinase-3 like protein 1 (CHI3L1). CHI3L1 interacts with interleukins to induce matrix metalloproteinase (MMP) expression, subsequently activating the MAPK signaling pathway (58). As a result, tumor cells can more readily disseminate through the stroma once ECM barriers are diminished.

A comprehensive understanding of the functions of SCs within the TME will deepen our knowledge of CRC metastasis and support the development of novel therapeutic strategies. Among emerging approaches, natural killer (NK) cell–based therapy has garnered substantial interest due to its potent anti-tumor activity (59). NK cells play a critical role in eliminating cancer cells and suppressing tumor growth and dissemination. Given their therapeutic potential in CRC, future research is expected to increasingly focus on strategies targeting the TME and modulating stromal components to enhance NK cell–mediated anti-tumor responses.

#### 2.1.3 ECM

The ECM is composed of a diverse array of molecules, including collagens, elastin, laminin, and fibronectin, all of which are essential for t issue architecture and remodeling (60). MMPs, although not structural ECM components, are critical enzymes that degrade ECM proteins, facilitating ECM turnover and restructuring—processes that are fundamental to tumor invasion and metastasis. The ECM provides structural support, influences cell signaling, and establishes the functional framework necessary for diverse cellular behaviors (61, 62). Through interactions with ECM components via surface receptors, tumor cells activate and release proteolytic enzymes that degrade the matrix, thereby promoting metastatic dissemination. Alterations in ECM composition and changes in the expression of cell surface receptors are frequently associated with the initiation, progression, invasion, and metastasis of malignant tumors (63).

Zhang et al. reported a significant increase in COL1A1 expression in CRC tissues and cell lines, which activates the WNT/PCP signaling pathway and enhances CRC metastasis (64). In patients with CRC and liver metastasis, collagen levels are elevated in both urine and plasma, with type IV collagen emerging as a promising biomarker for detecting liver metastasis (65, 66). Additionally, type I collagen within tumor tissues has been shown to promote EMT in CRC cells through activation of the integrin α2β1–PI3K/AKT/Snail signaling axis (67, 68). This pathway leads to the downregulation of E-cadherin, thereby facilitating tumor growth and increasing the likelihood of distant metastatic spread (69).

Tissue inhibitors of metalloproteinases (TIMPs) play a critical role in regulating tumor invasion, progression, and metastasis in CRC (70). MMP-1, expressed by fibroblasts, hepatocytes, and tumor cells, remains elevated even without external stimulation and serves as a key predictor of hepatic metastasis in CRC (71–73). The type IV collagenases MMP-2 and MMP-9 are closely associated with CRC progression, angiogenesis, and metastasis, and elevated serum levels of these enzymes provide greater diagnostic sensitivity than traditional markers such as CEA and CA19-9 (70, 74–76).

Given the ECM’s crucial role in CRC tumorigenesis and metastasis, it emerges as an important target for therapeutic interventions. Elevated expression of ECM biomarkers is associated with increased tumor invasion, metastasis, and poor clinical outcomes in CRC. Although ECM-targeted diagnostic and therapeutic strategies remain under development, the ECM offers substantial potential for anti-metastatic therapies and presents significant opportunities for future research.

#### 2.1.4 Exosomes

Exosomes are lipid bilayer vesicles found in various biological fluids and play a critical role in intercellular communication by transporting RNAs and proteins (77). Numerous studies have documented a wide array of miRNAs, mRNAs, lncRNAs, and proteins within these vesicles (**Table 1**) (78). Emerging evidence indicates that the RNA and protein cargo of exosomes exhibit distinct expression patterns in cancer, influencing tumor development. Min et al. conducted a comprehensive analysis of plasma-derived exosomal RNAs from CRC patients and identified specific biomarkers through RNA sequencing. Notably, three miRNAs—let-7b-3p, miR-139-3p, and miR-145-3p—were significantly enriched in these exosomes. The combined profiling of these miRNAs achieved an AUC of 0.927, outperforming total plasma miRNAs, which had an AUC of 0.707, in distinguishing CRC patients (79).

**Table 1:** Functions of exosomes as potential biomarkers in CRC.

NcRNA	Molecules	Expression	Function	References
miRNA	miR-23a	up	Increased in CRC cell line-derived exosomes	(234)
	miR-1246	up	Increased in CRC cell line-derived exosomes	(234)
	miR-125a-3p	up	Used for early diagnosis	(235)
	miR-1229	up	CRC diagnosis and lymph node metastasis	(236)
	miR-92a-3p	up	Lung metastasis	(237)
	miR-106b-3p	up	Lung metastasis	(238)
	miR-25-3p	up	Liver and lung metastasis	(239)
	miR-548c-5p	down	Liver metastasis	(240)
	miR-193a	up	Liver metastasis	(241)
	miR-130a	down	Inhibit the expression of HIF-1α	(242)
	miR-515-5p	down	Reduce the level of CXCL6	(243)
	miR-193a	down	CRC progession	(244)
lncRNA	lncRNA GAS5	up	Increased in CRC tissue	(245)
	lncRNA CCAT2	up	Increased in CRC tissue	(246)
	H19	up	Transcriptional regulation; miRNA sponging	(247)
	lncRNA CCAL	up	Increased in CRC tissues	(248)
	PCAT6	up	Combine with miR-330-5p	(249)
	JHDM1D-AS1	UP	Combine with DHX15	(250)

Exosomal miRNAs associated with cancer have been implicated in CRC metastasis. For instance, elevated levels of miR-203 in serum exosomes serve as an independent prognostic marker of poor outcomes in CRC patients (80). This oncogenic miRNA may promote liver metastasis by activating TAMs within the CRC microenvironment. Conversely, suppression of tumor-suppressive exosomal miRNAs can facilitate CRC metastasis. Reduced serum levels of miR-548c-5p, for example, are linked to unfavorable prognosis (81), whereas higher levels are associated with decreased CRC cell proliferation; conversely, its downregulation promotes cell proliferation and supports liver metastasis (82, 83). Additionally, significant reductions in serum exosomal miR-638 have been observed in CRC patients with liver metastasis (84).

These findings highlight the critical role of exosomes in the metastatic spread of CRC, particularly to the liver, which is the most frequent site of metastasis in clinical settings. The differential levels of RNAs and proteins within exosomes offer a promising avenue for identifying biomarkers and elucidating the molecular mechanisms underlying CRC metastasis.

### 2.2 EMT, cancer stem cells, and autophagy in CRC metastasis

EMT refers to the conversion of epithelial cells into cells exhibiting mesenchymal phenotypes. This transition is a crucial driver of tumor metastasis and significantly contributes to cancer progression (85, 86). A primary outcome of EMT is its role in enhancing anoikis resistance, a critical determinant of metastatic potential. Proteins promoting EMT act as key mediators of this resistance (87–89). Central to this mechanism are alterations in cell adhesion molecules; specifically, the reduced expression of E-cadherin accompanied by increased expression of N-cadherin, facilitating anoikis resistance, immune evasion, and enhanced metastatic capacity (90). Furthermore, EMT-associated transcription factors, such as Twist, Snail, and Zeb1, regulate E-cadherin and N-cadherin expression, thereby reinforcing anoikis resistance (91, 92). Multiple signaling pathways modulate EMT and its downstream effects on metastasis. Notably, inhibiting EMT using repurposed drugs, including simvastatin, an FDA-approved medication for hyperlipidemia, demonstrates promising therapeutic potential against CRC metastasis (93, 94). Thus, deeper exploration into EMT’s specific roles in CRC could offer valuable insights, guiding targeted therapeutic strategies.

Complementary to EMT, CSCs constitute a distinct tumor subpopulation critical for tumor initiation, growth, metastasis, and therapeutic resistance. Characterized by specific surface markers such as CD44, CD24, CD29, and epithelial-specific antigen (ESA), CSCs have a unique capability to migrate to distant organs and initiate secondary tumors, emphasizing their pivotal role in the metastatic cascade (95–98). However, considerable research gaps persist. Further clarity distinguishing CSCs from normal stem cells is required, along with the identification of therapeutic targets capable of selectively eliminating CSCs without impairing normal stem cell function.

Autophagy serves dual roles in cancer biology. Under physiological conditions, autophagy maintains cellular homeostasis by facilitating the turnover of cellular components. Under adverse conditions such as nutrient deprivation, oxidative stress, or hypoxia, it promotes survival through recycling cellular components (99, 100). Emerging evidence indicates autophagy also contributes to tumor metastasis. Tumor cells in advanced stages often activate autophagy in response to various stressors, thereby enhancing their proliferation and metastatic capacity (101). Thus, therapeutic strategies targeting autophagy, especially those disrupting autophagosome–lysosome fusion, such as chloroquine, hydroxychloroquine, and the chloroquine derivative Lys05, represent critical approaches, inhibiting a pivotal step in the autophagic pathway and thus hindering metastatic progression (102, 103).

### 2.3 Angiogenesis and lymphangiogenesis

Angiogenesis and lymphangiogenesis, processes involving the formation of new blood and lymphatic vessels, respectively, are essential for the proliferation and dissemination of primary and metastatic tumors. These vascular networks facilitate nutrient delivery to tumors and provide routes for metastatic spread. In CRC, the tumor stroma, consisting primarily of CAFs and immune cells, plays a pivotal role in promoting angiogenesis. Elevated expression of Wingless-type MMTV integration site family member 2 (WNT2) has been observed in CRC, facilitating tumor cell invasion and dissemination (35). Moreover, microRNAs significantly regulate angiogenesis; specific microRNAs, including miR-194, miR-17-92, and miR-126, modulate vascular development by targeting critical genes (104–106).

Similarly, lymphangiogenesis significantly influences CRC progression, particularly regarding lymph node metastasis (107–109). Essential lymphangiogenic factors, notably VEGF-C and VEGF-D, promote lymphatic dissemination, whereas VEGF-A predominantly drives angiogenesis (63, 110, 111). Elevated VEGF-D expression in CRC often correlates with poor clinical outcomes. Clinical trials involving metastatic CRC (mCRC) demonstrate that targeting VEGF-A with bevacizumab results in increased VEGF-D levels, potentially explaining the limited efficacy of bevacizumab therapy in certain patients (112, 113). A thorough understanding of the interplay between angiogenesis and lymphangiogenesis in CRC metastasis is crucial for developing effective targeted therapies.

## 3 Research on common metastatic sites of CRC

### 3.1 CRC liver metastasis

Approximately 25% of individuals diagnosed with primary CRC present with liver metastases at diagnosis, and nearly 50% eventually develop liver metastases during disease progression (114). Despite millions of cancer cells disseminating via portal circulation, liver metastasis is characterized by relatively low colonization efficiency, with few cells successfully proliferating within liver tissue (115). Research underscores the importance of the TME and associated regulatory factors, including immune modulators, metabolic reprogramming, intercellular interactions, genomic alterations, and gut microbiome influences, in CRC liver metastasis. Studies indicate that immune regulatory molecules such as programmed death-1 (PD-1) and programmed death-ligand 1 (PD-L1) correlate with unfavorable outcomes and immune tolerance by promoting regulatory T cell (Treg) activation in CRC and gastric cancer (114). CRC-derived liver metastases exhibit increased PD-L1 expression compared to primary tumors, possibly explaining diminished responsiveness to PD-1 blockade therapies (116–118). Furthermore, TAMs facilitate tumor cell migration and EMT within the TME (119, 120). Within the liver, macrophages differentiate into M1 or M2 subtypes in response to various cytokines, with M2 macrophages predominantly promoting tumor progression through angiogenesis and invasion (121–123). The heightened metabolic demands of tumor cells frequently create a TME characterized by hypoxia, acidity, and nutrient scarcity, conditions that activate lipid metabolism pathways. Lipid metabolites subsequently enhance tumor progression and add complexity to the TME (124, 125). Furthermore, upregulated aldolase B (ALDOB) enhances fructose metabolism, providing an alternative energy source for proliferative growth in CRC liver metastases (126, 127). Yang et al. reported that macrophages expressing metastasis-associated CD36 uptake tumor-derived extracellular vesicles enriched with long-chain fatty acids, acquiring protumor characteristics in preclinical liver metastasis models (128). Loo et al. demonstrated that miR-551a and miR-483 function as natural inhibitors of CRC liver colonization and metastasis by selectively targeting brain-type creatine kinase (CKB) secreted from hypoxic metastatic liver cells, thus facilitating ATP synthesis and supporting metastatic cell survival (129).

Metastatic organotropism, the preferential metastasis to specific organs, depends on intrinsic tumor-cell properties and interactions with local molecular and cellular elements of the microenvironment. Prior to tumor cell dissemination, hepatocytes release factors that attract and activate immune and stromal cells, thereby establishing a pre-metastatic niche. Genomic studies reveal that CRC-derived peptidylarginine deiminase 4 (PAD4) facilitates liver metastasis through ECM citrullination. Inhibiting PAD4 modifies EMT markers and reduces metastatic potential (130, 131). Moreover, the gut microbiome significantly influences metastatic processes and pre-metastatic niche formation. CRC-associated microbiota promote tumor growth via toxins that damage host DNA and activate signaling pathways including STAT3, NF-κB, Wnt, and SREBP-2, thereby inducing COX-2 expression and creating an inflammatory microenvironment enriched with reactive oxygen species (ROS) and reactive nitrogen species (RNS), ultimately enhancing immune evasion and metastatic potential (132, 133). Disrupted tight junctions in CRC activate YAP signaling, suppressing FOXD3 expression and consequently reducing activity of the m6A methyltransferase METTL3, leading to increased expression of kinesin family member 26B (KIF26B) and promoting metastasis (134). Although not fully elucidated, interactions between host immunity and gut microbiome are increasingly recognized as crucial for CRC metastasis, representing a significant area for future research to develop novel preventive and therapeutic approaches.

### 3.2 CRC lung metastasis

The lung is a frequent metastatic site for CRC, occurring in approximately 20–30% of cases (135). Evidence suggests that lung metastases predominantly arise through hematogenous rather than lymphatic dissemination. Tumors originating from the lower rectum, specifically, often bypass the portal venous system by entering the inferior rectal vein, enabling rapid pulmonary colonization (136, 137). Guillemot et al. demonstrated that activation of CXCR7 (rather than CXCR4) on tumor-associated vasculature significantly enhances CRC dissemination to the lungs (138). As CRC progresses, the SMAD4 complex, a critical tumor suppressor in the TGF-β signaling pathway, translocates into the nucleus. Immunohistochemical (IHC) analysis of pulmonary metastases reveals a significant correlation between SMAD4 loss and increased expression of CCL15, with metastatic lesions expressing CCL15 nearly doubling those without its expression. Wang et al. reported that knockdown of c-MYC substantially enhances the inhibitory effects of arenobufagin on the Nrf2 signaling pathway, as well as on CRC cell migration and invasion, highlighting the importance of disrupting the c-MYC/Nrf2 axis to mitigate lung metastasis (139). Continued advances in molecular biology are anticipated to clarify mechanisms underlying CRC lung metastasis and foster novel therapeutic strategies, potentially improving patient survival significantly.

### 3.3 CRC peritoneal metastasis

PM occurs in approximately 25% of CRC patients (140). Zajac et al. observed that peritoneal dissemination and collective invasion are promoted by cancer spheroids exhibiting outward apical polarity (141), facilitating malignant cell movement within the peritoneal cavity. Recent studies indicate that TAMs promote tumor growth and metastasis by enhancing angiogenesis, establishing pre-metastatic niches, and inducing immunosuppression. Inflammatory mediators from tumors activate omental neutrophils, prompting them to release protein-coated chromatin, forming localized traps that serve as pre-metastatic niches (142). Ubink et al. analyzed histopathological and molecular characteristics of primary CRC and associated peritoneal metastases, demonstrating that peritoneal metastases typically exhibit reduced stromal content compared to 79% of primary tumors (143). Clinically, peritoneal surgical trauma frequently leads to adhesions. However, minimally invasive laparoscopic surgery using warm, humidified CO_2_ insufflation, rather than cold, dry CO_2_, reduces expression of cyclooxygenase-2 (COX-2), vascular endothelial growth factor (VEGF), and hypoxia-inducible factor 1α (HIF-1α), thus diminishing tumor cell implantation and proliferation (144–146). Moreover, Berkovich et al. serially analyzed peritoneal drainage fluid post-colon surgery, finding that collected fluid enhances colon cancer cell migration *in vitro (*147).

### 3.4 CRC bone metastasis

CRC bone metastases are relatively uncommon, occurring in fewer than 10% of patients (148–150). These metastases are predominantly osteolytic, with osteoclasts being the primary target cells (151). It is hypothesized that bone tropism originates within the primary tumor. Evidence indicates that CAFs create a microenvironment resembling bone marrow, selectively favoring cancer cells adapted for bone colonization. Bone tissues abundantly express chemokines such as CXCL12, while CRC cells frequently express the receptor CXCR4, guiding migration to bone sites (152, 153). The bone microenvironment, rich in blood vessels, growth factors, and interleukins, provides an optimal niche for rapid tumor cell expansion. Beyond ligand-receptor interactions, osteoclast activity is regulated by signaling pathways including MAPK and PI3K/AKT (154, 155). CRC cells produce RANKL, which interacts with the receptor RANK on osteoclast precursors and mature osteoclasts (156), further promoting bone metastasis (157). Unlike pulmonary metastases, bone microenvironments are typically hypoxic (158). In CRC, elevated lactate production activates the p38/MAPK signaling pathway, fostering tumor progression and upregulating VEGF expression, thus creating a pre-metastatic niche in distant sites such as bone (159, 160).

### 3.5 CRC brain metastasis

Brain metastasis in CRC indicates disruption of the blood-brain barrier (BBB), a critical physiological safeguard. Studies have demonstrated that CRC cells secrete proteases, particularly MMPs, which degrade the basement membrane and ECM components at the BBB, disrupting endothelial tight junctions and thereby increasing barrier permeability (161–163). MMP-2 and MMP-9, known to target type IV collagen and basal membrane constituents, significantly enhance tumor cell invasion. Once CRC cells breach the BBB, the barrier poses a major therapeutic challenge, as most chemotherapeutic agents fail to effectively cross it (164). Scartozzi et al. observed discordances in EGFR status between primary CRC tumors and their brain metastases, identifying EGFR positivity in 15% of metastases originating from initially EGFR-negative primary tumors (165). Additionally, brain metastases exhibit higher frequencies of KRAS mutations, indicating a potential role for KRAS signaling in CRC brain metastasis progression (166, 167). Furthermore, microRNAs (miRNAs) play essential roles in metastasis. Li et al. noted alterations in miRNA expression within brain metastases, observing downregulation of certain miRNAs and upregulation of others (168). Nishioku et al. demonstrated that S100A4 promotes p53 expression in brain microvascular endothelial cells, thus compromising BBB integrity. Elevated S100A4 levels correlate with advanced tumor stages and increased incidence of CRC brain metastases, suggesting a significant role in metastatic progression (169, 170).

Despite recent advances, mechanisms underpinning CRC brain metastasis remain incompletely understood, necessitating further investigation. Continued research into these molecular pathways is essential to enhance diagnosis and therapeutic strategies for CRC brain metastasis.

### 3.6 Paired studies of primary and metastatic lesions

The analysis of paired primary and metastatic CRC lesion samples provides crucial insights into metastatic mechanisms, guiding targeted therapeutic interventions. Studies examining the relationship between metastasis and telomere length found no significant difference between primary tumors and liver metastases. However, patients with metachronous hepatic metastases demonstrated shorter telomeres compared to those with synchronous metastases (171). Lee et al., employing next-generation sequencing (NGS), identified KRAS mutations in 55% of peritoneal metastasis cases and TP53 mutations in 36% of these samples (172). Mao et al. compared genomic profiles of primary CRC tumors with their metastases, noting high concordance in common driver mutations (KRAS, BRAF, and PIK3CA). However, PTEN loss was inconsistent across samples. Hepatic metastases displayed higher genomic similarity to their primary tumors compared to lymph node metastases, highlighting mutation profile variability across metastatic sites (173). This emphasizes the importance of further genomic studies across diverse metastatic locations to elucidate CRC progression and optimize therapeutic strategies.

**Figure 2:**
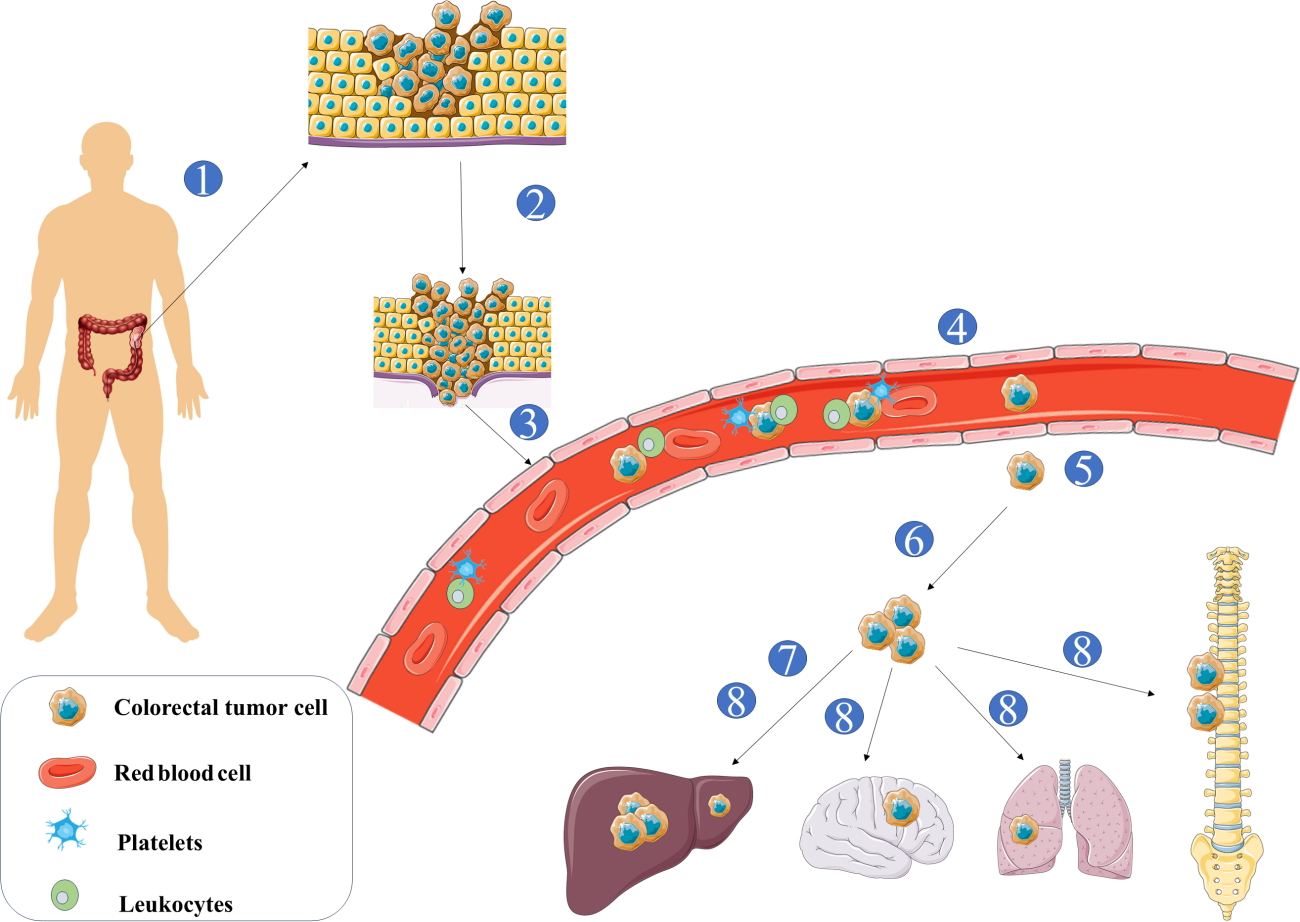
① Tumor cells leave the original organ ②Tumor cells invade the ECM via basement membrane degradation ③Tumor cells induce angiogenesis and infiltrate the circulation ④ Tumor cell survival in circulation relies on clustering with themselves, leukocytes and platelets. ⑤Tumor cells surviving circulation exit blood into the target organ’s stroma. ⑥Tumor cells seed into the secondary site.⑦Tumor cells enter dormancy ⑧Tumor cell reactivation facilitates their outgrowth and colonization at the secondary site.

These paired studies are essential for identifying critical metastasis-associated genes and understanding gene expression heterogeneity between primary tumors and metastatic lesions. Such insights are crucial for advancing diagnostic accuracy and therapeutic effectiveness in CRC metastasis (**Figure 2**).

## 4 Predictive models for CRC and metastasis

### 4.1 Animal models

Animal models are indispensable tools in oncology research, providing essential platforms for investigating CRC pathogenesis, metastatic mechanisms, and therapeutic efficacy. Numerous murine models have been developed to simulate CRC initiation, progression, and treatment responses observed in humans. In 1969, Rygaard and Povlsen significantly advanced this field by establishing patient-derived xenograft (PDX) models, created by transplanting human tumors into immunodeficient mice (174, 175). Similarly, cell line-derived xenograft (CDX) models are generated by implanting cultured tumor cells into immunodeficient hosts (176, 177).

Norihiko et al. demonstrated that different injection sites in animal models replicate distinct metastatic processes reflecting the specific characteristics of target tissues. For example, stereotactic brain injection models facilitate observation of tumor proliferation at injection sites and subsequent infiltration into brain parenchyma. Conversely, intrathecal injection models accurately mimic leptomeningeal carcinomatosis, whereas intracarotid injection models represent hematogenous metastasis by avoiding perivascular and invasive proliferation. Although immunodeficient mouse models can mimic human-specific immune and stromal microenvironments, their pharmacological responses often differ from clinical observations. Follain et al. confirmed heterogeneity in blood flow characteristics among metastatic organs, noting, for instance, capillary flow rates of approximately 628 μm/s in the brain. Consequently, clinical studies require targeted optimization strategies: drug concentrations capable of crossing the BBB should be increased in brain metastasis trials, and drug administration frequency might be adjusted according to portal vein blood flow in liver metastasis studies. Given that CTC clusters exhibit higher metastatic potential, they can serve as patient stratification markers. Thus, therapeutic strategies employing “anti-adhesion and anti-aggregation” approaches, combining integrin inhibitors and platelet inhibitors, could be optimized based on adhesion mechanics (178). McGovern et al. enhanced these models by incorporating human CAFs, lymphatic/endothelial cells, and humanized tissue-engineered bone constructs (179). These enhanced xenograft models responded to treatments such as zoledronic acid but failed to respond to denosumab (180), differing from clinical trials where denosumab demonstrated superior efficacy (181, 182).

Recent technological advances have expanded beyond conventional animal models. Microfluidic three-dimensional (3D) tissue chip platforms serve as critical intermediaries between two-dimensional co-cultures and *in vivo* studies, offering improved representations of metastatic niches (183, 184). The interaction between tumor and endothelial cells, an essential yet frequently overlooked element in metastatic niche simulations, is crucial for exploring malignant angiogenesis and the development of anti-angiogenic therapies for solid tumors (185, 186). Meng et al. employed 3D bioprinting methods to generate *in vitro* tumor models that closely replicate key characteristics of the TME, including malignant angiogenesis (187).

While murine models offer valuable insights into CRC pathogenesis, metastasis, and therapeutic responses, they cannot entirely replicate the complexity of human CRC progression. Consequently, refined modeling techniques are necessary to accurately simulate CRC metastasis *in vivo* and enhance diagnostic and therapeutic innovations.

### 4.2 Clinico-AI predictive models

Artificial intelligence (AI) encompasses technologies designed to emulate human intelligence and capabilities. In healthcare, where large amounts of data are continuously generated, well-designed and adequately trained AI systems can outperform human clinicians regarding accuracy, efficiency, and reduction of biases in certain tasks. AI models that integrate qualitative and quantitative data from medical imaging (radiomics) and clinical records can uncover intricate patterns and correlations within extensive datasets. These models enhance predictive accuracy by combining clinical indices, pathological data, and molecular biomarkers, thereby supporting evidence-based clinical decision-making (**Table 2**) (188).

**Table 2:** Application of artificial intelligence in tumor metastasis.

Ref.	Imaging	Main aim	Patients (n)	Main fingdings
Li et al. (251)	CT	Prediction of nodes metastases	766	AUC=0.750 Specificity =0.843
Taghavi et al. (252)	CT	Prediction of synchronous liver metastases	91	The radiomics model outperformed the clinical model: AUC = 0.93 VS 0.64
Rao et al. (253)	CT	Prediction of synchronous liver metastases	29	AUC = 0.73-0.78
Jing et al. (254)	CT	Prediction of liver metastases	212	The fusion model performance: AUC = 0.761
Liu et al. (255)	MRI	Prediction of distant metastasis	235	Model performance: C-index=0.747 AUC = 03894
Liu et al. (256)	MRI	Prediction of synchronous liver metastases	169	Radiomics and nomogram:AUC=0.866 and 0.944
Liu et al. (257)	MRI	Prediction of nodes metastases	186	Clinical-radiomics model improves performance: AUC = 0.827
Shu et al. (258)	MRI	Prediction of synchronous liver metastases	194	The Radiomics model combined clinical performance:AUC=0.921
Liu et al. (259)	MRI	Prediction of synchronous distant metastasis	177	The clinical-radiomics combined model performance:AUC=0.827
He et al. (260)	PET/CT	Prediction of nodes metastases	199	AUC=0866 and 0.903

In predicting CRC liver metastasis (CRCLM), key clinical variables such as age, sex, CEA, CA19-9, and tumor stage (T and N stages), recognized primary predictors, can be augmented with radiomics-derived imaging features. The random forest (RF) algorithm is commonly utilized for this task, given its capability to integrate predictions from multiple decision trees and manage data without extensive preprocessing. Its intrinsic feature-selection ability addresses the “curse of dimensionality” inherent in high-dimensional data, uncovering hidden insights. Moreover, its parallelizable structure enables rapid training on large datasets, reducing overfitting and enhancing model generalizability. Hao et al. developed a CRCLM risk prediction nomogram achieving a C-index of 0.787, demonstrating reliability through calibration and ROC analyses (AUC = 0.784). Incorporating clinical parameters into the nomogram visually illustrates each predictor’s influence, enabling personalized CRCLM risk predictions. Combining SVM, RF, and Cox regression nomograms has shown superior predictive performance compared to LR and CNN methods. Specifically, SVM effectively manages complex, high-dimensional data and captures nonlinear relationships; RF integrates multiple decision trees to minimize overfitting; Cox regression nomograms utilize clinical data to generate individualized risk assessments (189, 190). Lee et al. reported that among 2,019 stage I–III CRC patients, integrating CT imaging features extracted via VGG16 with PCA-based dimensionality reduction (PC1) and clinical variables provided the most accurate five-year liver metastasis predictions (AUC = 0.747), surpassing the clinical-only model (AUC = 0.709). PC1-based classification correlated significantly with survival (P < 0.01); however, imaging features alone did not enhance mortality prediction (191).

To further refine predictive precision regarding tumor responses to pharmacotherapies, recent AI advancements include the Radiopathomics Integrated Predictive and Diagnostic System (RAPIDS), developed by Feng et al. RAPIDS provides a robust and precise modeling framework, serving as a pioneering tool in personalized cancer management (192).

## 5 Clinical management of CRC metastasis

Achieving curative outcomes in metastatic CRC (mCRC) remains highly challenging. Despite incremental progress, innovative therapeutic approaches are urgently required, prompting extensive clinical research into personalized treatment strategies. As previously addressed in this work, CRC commonly metastasizes to specific organs, necessitating tailored therapeutic strategies across these diverse sites. Advances in these areas could establish groundbreaking therapeutic paradigms (**Table 3**).

**Table 3:** Drug therapy for CRC metastases.

Classification	Drug	Mechanism of action	Use in mCRC	Reference
Systemic chemotherapy	Fluorouracil (5-FU)	Inhibits formation of thymidylate from uracil	All lines of therapy	(261)
	Irinotecan Hydrochloride	Topoisomerase I inhibitor	All lines of therapy	(205)
	Oxaliplatin	Forms intrastrand DNA adducts	All lines of therapy	(196)
	Capecitabine	Pro-drug of 5-FU; inhibits formation of thymidylate from uracil	All lines of therapy	(262)
Targeted therapy	Bevacizumab	VEGF inhibitor	Any line of therapy in combination with 5-FU, irinotecan, and/or oxaliplatin	(224)
	Cetuximab	EGFR inhibitor	In *EGFR* mutant, *RAS/RAF* wild-type cancers;	(263)
	Panitumumab	EGFR inhibitor	In *EGFR* mutant, *RAS/RAF* wild-type cancers; any line of therapy in combination with 5-FU, irinotecan, and/or oxaliplatin	(264)
	Regorafenib	Multi-kinase inhibitor	Second-line therapy or beyond	(265)
Immunotherapy	Nivolumab	PD-1 inhibitor	In high MSI or dMMR cancers	(266)
	Ipilimumab	CTLA-4 inhibitor	In high MSI or dMMR cancers	(267)
	Pembrolizumab	PD-1 inhibitor	In high MSI or dMMR cancers	(268)

Liver metastases are surgically resectable in only 20–30% of cases (193), with recurrence rates exceeding 50% post-resection (194, 195). Three primary strategies have emerged: neoadjuvant chemotherapy (e.g., FOLFOX or CAPOX regimens) prior to liver surgery, perioperative chemotherapy (pre- and post-operative FOLFOX/CAPOX-based protocols), and initial systemic chemotherapy combined with biologics followed by surgical resection (195). In many regions, systemic chemotherapy including oxaliplatin or irinotecan remains standard care for CRCLM patients (196). Such chemotherapy reduces lesion size, rendering 12–54% of initially inoperable cases suitable for resection (197). Monotherapy with immunotherapy typically produces limited outcomes in liver metastases; however, combining immunotherapy with chemotherapy demonstrates significant promise (198). For resected CRC patients with MSI-H/dMMR tumors, oxaliplatin-based adjuvant therapy improves prognosis and therapeutic efficacy (196, 199, 200). A novel therapeutic strategy currently under investigation involves fecal microbiota transplantation combined with repeated anti-PD-1 administration (NCT04729322; n = 15) for MSI-H/dMMR mCRC patients previously resistant to initial anti-PD-1 treatment (201).

Compared to liver metastases, the biological mechanisms and TME driving lung metastasis in CRC are less understood (202). Pharmacotherapy is routinely applied; however, evidence supporting chemotherapy’s effectiveness, with or without targeted agents, remains limited. Reported objective response rates (ORR) for first-line chemotherapy ± targeted therapy are below 25%, significantly lower than previous reports of 35% (203, 204). Lung metastases tend to exhibit a broader spectrum of genetic mutations and a higher tumor mutational burden (TMB) than liver metastases, highlighting the potential for molecular biomarker-guided immunotherapies and novel therapeutics targeting lung-specific genetic drivers (203).

In managing CRC peritoneal metastasis (PM), hyperthermic intraperitoneal chemotherapy (HIPEC) with agents such as oxaliplatin, pegylated liposomal doxorubicin, paclitaxel, and carboplatin is widely utilized. However, clinical application of intraperitoneal irinotecan is limited due to significant hematologic toxicity (205). Franko et al. demonstrated significantly prolonged median overall survival (mOS) in the HIPEC-treated group (206). Most synchronous peritoneal metastases occur concurrently with hematogenous dissemination, and retrospective multicenter studies identify R0 resection as a positive prognostic indicator (207–209). Aggressive cytoreductive surgery (CRS) combined with HIPEC significantly improves overall survival (210–212).

Clinical complications of CRC bone metastasis, including spinal cord compression, pathological fractures, and hypercalcemia, necessitate interventions targeting osteoclast activity. Common treatments include bisphosphonates (e.g., zoledronic acid) and denosumab (213). These agents inhibit osteoclast proliferation and induce apoptosis (214). Denosumab specifically interacts with the receptor activator of nuclear factor-kappa B (RANK) ligand, preventing osteoclast maturation (215). Alternative strategies involve enhancing bone formation through modulation of SCs and osteoprogenitor cells in the bone microenvironment (216–218). Immunotherapy can further reduce bone resorption by amplifying anti-tumor CD8+ T-cell and NK-cell responses, enhancing tumor immune surveillance (218). Additionally, electrochemotherapy (ECT), a minimally invasive approach combining high-voltage electric pulses with anticancer agents, effectively treats bone metastases while preserving bone mineral integrity and regenerative capacity. Notably, bleomycin and cisplatin demonstrate efficacy when used alongside ECT (218–220).

Brain metastases (BM) in CRC represent a considerable clinical challenge, generally arising during advanced disease stages and frequently remaining asymptomatic. Current guidelines recommend systemic chemotherapy for symptomatic BM patients. However, treatment efficacy is limited by the poor penetration of chemotherapeutic agents across the BBB. Recent studies suggest that bevacizumab, by targeting vascular endothelial growth factor (VEGF) within the brain without requiring BBB penetration, may represent an effective therapeutic strategy for CRC-related BM (221, 222). A study by Li et al. involving 21 CRC BM patients indicated a potential role for bevacizumab, although results did not reach statistical significance (223). Additionally, a case report by Hung Van Nguyen et al. described symptom resolution in a CRC BM patient only after treatment with bevacizumab (224). Another treatment option is temozolomide, an alkylating agent capable of crossing the BBB, which sensitizes malignant cells to radiotherapy (223). Future research should focus on novel therapeutic agents aimed at improving survival outcomes for CRC BM patients.

Despite significant advances in CRC treatment in recent years, many patients still do not benefit from targeted and immune therapies due to the absence of actionable mutations or dMMR status, resulting in limited therapeutic options. Emerging approaches, such as immune cell engineering and nanotechnology, offer promising therapeutic strategies for these patients. CAR-T cell therapy involves extracting and genetically modifying patients’ T cells to express CARs, enabling them to target and eliminate cancer cells following reinfusion. The application of healthy donor T cells is restricted by toxicity, notably GVHD; however, preclinical studies have successfully circumvented this issue using CRISPR/Cas9-mediated ablation of donor TCRs (225). While effective in hematological malignancies, CAR-T therapy has demonstrated response rates of only 9% in solid tumors, including CRC (226). The primary limitation is a lack of tumor-specific target antigens. Currently, no phase III clinical trials evaluate CAR-T cells in advanced CRC. Nevertheless, early-phase trials and preclinical studies indicate potential for optimized CAR-T therapies targeting antigens such as TAG-72, GUCY2C, CEA, combinations involving CD30, and HER2 (227, 228). Nanotechnology also provides effective drug delivery platforms for anticancer agents. Preclinical studies in 2018 demonstrated that nanoparticle-encapsulated 5-FU enhanced chemotherapy efficacy and reduced adverse effects in breast cancer models (229). Recent research has confirmed targeted nanocarriers’ capability to deliver drugs specifically to mCRC. In 2021, GCC-targeted nanocarriers encapsulating etoposide specifically inhibited metastatic lesion growth in mouse models. Nanoparticles can similarly deliver miR-122 (230). In liver mCRC models, this approach holds promise for enhancing anti-tumor T-cell activity, preventing metastasis, prolonging survival, and achieving synergy with immunotherapy or targeted treatments, representing a novel therapeutic direction for mCRC.

## 6 Conclusions

The mechanisms underlying CRC metastasis are intricate and involve multiple factors that significantly influence patient outcomes. A comprehensive understanding of these mechanisms is essential for improving clinical diagnosis and therapeutic strategies. The CRC TME constitutes a dynamic and heterogeneous milieu actively contributing to metastatic progression (231). Environmental conditions, such as reduced pH and hypoxia within the TME, promote cancer cell dissemination and metabolic reprogramming (232). Stromal components, including CAFs and immune cells, significantly enhance CRC cell invasion and migration. Additionally, ECM remodeling and degradation mediated by MMPs are critical steps in the metastatic cascade. Moreover, processes such as EMT, angiogenesis, and lymphangiogenesis play essential roles in CRC dissemination. Collectively, these TME factors elucidate the initiation, progression, metastasis, and therapeutic opportunities in CRC, some of which are already clinically implemented. Nevertheless, ongoing research remains crucial to evaluate adverse effects of emerging treatments and to explore newly identified factors contributing to CRC metastasis. In-depth investigation of these factors and their genetic basis will likely guide future research efforts.

CRC exhibits organ-specific metastatic preferences, characterized by distinct molecular drivers and therapeutic strategies for metastases in the liver, lungs, peritoneum, and brain (233). Although treatment modalities vary, mechanisms specific to each metastatic site remain inadequately understood, often resulting in suboptimal therapeutic outcomes. An improved understanding of factors governing organ-specific metastasis may identify novel targets for personalized treatment strategies. Additionally, developing preclinical models that accurately recapitulate human CRC metastasis remains a significant ongoing challenge. Despite considerable advancements, substantial gaps persist in the understanding of CRC metastasis. Translating these mechanistic insights into universally effective clinical interventions remains a long-term goal. Nevertheless, continued scientific investigation is anticipated to clarify the complex biology of CRC metastasis, ultimately providing renewed hope for patients.
